# Socioeconomic status and time in glucose target range in people with type 2 diabetes: a baseline analysis of the GP-OSMOTIC study

**DOI:** 10.1186/s12902-018-0279-6

**Published:** 2018-07-21

**Authors:** Mei Lyn Tan, Jo-Anne Manski-Nankervis, Sharmala Thuraisingam, Alicia Jenkins, David O’Neal, John Furler

**Affiliations:** 1 Department of General Practice, University of Melbourne, Level 1, 200 Berkeley St, Carlton, VIC 3010 Australia; 2 NHMRC Clinical Trials Centre, University of Sydney, Levels 4-6 Medical Foundation Building, 92-94 Parramatta Rd, Camperdown, NSW 2050 Australia; 3 Department of Medicine, St Vincent’s Hospital, The University of Melbourne, Level 4, Clinical Sciences Building, 29 Regent St, Fitzroy, VIC 3065 Australia

**Keywords:** Type 2 diabetes mellitus, Primary care, Continuous glucose monitors, Time in glucose target range, Socioeconomic status

## Abstract

**Background:**

Optimal glycaemia, reflected by glycated haemoglobin (HbA1c) levels, is key in reducing type 2 diabetes (T2D) complications. However, most people with T2D have suboptimal recall and understanding of HbA1c. Continuous glucose monitoring (CGM) measures glucose levels every 5 to 15-min over days and may be more readily understood. Given that T2D is more common in lower socioeconomic settings, we aim to study relationships between socioeconomic status (SES) and percentage time in glucose target range (TIR) which is a key metric calculated from CGM.

**Methods:**

Analysis of baseline data from the General Practice Optimising Structured MOnitoring To Improve Clinical outcomes (GP-OSMOTIC) randomised controlled trial (October 2016 – November 2017) of 300 people with T2D from 25 Victorian General Practices. *FreeStyle Libre Pro®* sensor patch was used for this study. SES was defined by the Index of Relative Socio-economic Disadvantage (IRSD) and educational attainment. Univariable and multivariable mixed-effects linear regression analyses controlling for age, BMI, diet, exercise and study arm were performed.

**Results:**

One hundred and sixty-seven (60.1%) participants were male, the mean (SD) participant age was 61.0 (9.7) years, and the mean (SD) duration of CGM use was 12.3 (2.5) days. The 10th IRSD decile (least disadvantaged) was associated with a 15% higher TIR vs. the 1st decile (most disadvantaged) (95% CI 5, 25; *p* = 0.003) and a 0.6% lower HbA1c (95% CI 0.1, 1; *p* = 0.03). There was no evidence of an association between educational attainment and TIR/HbA1c.

**Conclusion:**

Higher SES measured at an area level is associated with better achievement of glycaemic target using complementary measures of HbA1c and TIR in the GP-OSMOTIC cohort. Given that TIR may be more easily used in patient education and self-management support compared to HbA1c values, the social gradient identified in TIR provides an opportunity for clinicians and policy makers to address health inequities in T2D.

**Trial registration:**

Australian and New Zealand Clinical Trials Registry Trial ACTRN12616001372471, prospective, Date registered 4/10/2016.

## Background

Approximately 1.2 million (5%) Australians are currently living with type 2 diabetes (T2D), increasing from 840,000 people (3.8%) in 2011–2012 [1, 2]. Strategies aimed at optimising blood glucose levels are a fundamental premise for the prevention and progression of complication in people with T2D. Current Australian guidelines set targets for glycated haemoglobin (HbA1c), an index of average blood glucose level over three months to assess glycaemia and as a measure of risk for the development of diabetes-related vascular and neurological complications [3]. Self-management (adherence to medications, managing diet and exercise) is an important way for people with T2D to achieve and maintain glycaemic targets.

Significant inequities exist with prevalence, mortality and hospitalisations all twice as common in people with T2D from low socioeconomic backgrounds compared to people from high socioeconomic backgrounds [1, 4]. Increasing levels of socioeconomic disadvantage are also associated with higher likelihood of out of target (high) HbA1c [5].

Research has shown that patients with diabetes have suboptimal levels of recall and understanding of HbA1c (only around 25% report a good understanding), which in turn impacts significantly on patients’ diabetes self-care behaviours [6–8]. This is particularly important in the setting of socioeconomic disadvantage which is associated with low levels of health literacy and education [9–11].

Continuous glucose monitoring (CGM) technology provides a level of detail when assessing glucose control not provided by HbA1c measurements. CGM measures interstitial glucose levels nearly continuously allowing insights into short and medium-term fluctuations in glucose levels. Several parameters can be derived from CGM data including a measurement of percentage of time in glucose target range (TIR), a measure of the amount of time an individual spends within a specified glucose target range. Previous studies have identified a correlation between TIR and HbA1c. Having 50% of self-monitored glucose levels within 3.9–10 mmol/l (70.2–180 mg/dL) is correlated with an HbA1c of around 7% [12–15]. TIR, dependent on fluctuations in diet and physical activity, medication and adherence, is known to be an independent predictor of diabetes complications and mortality [13, 16].

Understanding the association between TIR and socioeconomic status (SES) may provide important insights in addition to those of HbA1c when evaluating the socioeconomic disparity seen in T2D prevalence and the development of complications. Importantly, TIR may also be a measurement that is easier for people with T2D to interpret and comprehend, empowering them to optimise their self-care behaviours. This is because TIR directly relates to measurements that are being made (i.e. blood glucose levels and a range), whereas HbA1c is one remove from that as it indirectly references blood glucose levels, therefore possibly making this harder conceptually for people to understand.

The General Practice Optimising Structured MOnitoring To Improve Clinical outcome (GP-OSMOTIC) randomised controlled trial is a study on the impact of a retrospective CGM device (*Abbott FreeStyle Libre Pro® Flash Glucose Monitoring System*) used in the clinical care of people with T2D in General Practice in Australia [17]. We report here on the relationship found between SES and TIR based on analysis of baseline data from the GP-OSMOTIC randomised controlled trial.

## Methods

### Design and study participants

This is an analysis of baseline data obtained from the GP-OSMOTIC trial (ACTRN12616001372471) that recruited a total of 300 patients from 25 General Practices in Victoria, Australia, between October 2016 and November 2017.

GP-OSMOTIC trial inclusion criteria are ages 18–80 years old, patients diagnosed with T2D, patients with the most recent (in the previous 3 months) HbA1c level 0.5% above their individualised target, patients prescribed at least 2 non-insulin anti-hyperglycaemic agents and/or insulin, and patients who have had stable anti-hyperglycaemic therapy for the last four months. Individualised target refers to an HbA1c target based on the participant’s clinical characteristics and risk profile.

GP-OSMOTIC trial exclusion criteria are patients with debilitating medical conditions (e.g. unstable CVD, severe mental illness, end-stage cancer), eGFR < 30 ml/min/1.73m^2^, proliferative retinopathy, patients who are pregnant/lactating/planning pregnancy, patients unable to speak English/give consent, patients unwilling to use CGM device or follow the GP-OSMOTIC study protocol, a history of allergy to plaster/tape and any condition that makes monitoring diabetes using the HbA1c unreliable (e.g. haemoglobinopathies).

### Baseline data

Baseline survey, anthropometric measures and pathology collection were undertaken by clinically-trained research assistants at each participant’s general practice. The survey included questions on educational attainment, employment and occupation, ethnicity and smoking status. Participant’s medications, co-morbidities and diabetes-related complications were retrieved from clinical medical electronic health records. Chronic kidney disease was present if participants had evidence of microalbuminuria/macroalbuminuria and/or eGFR of < 60 mL/min/1.73 m^2^ from urine and blood samples performed up to 30 days prior to baseline. eGFR was calculated by pathology labs using the Chronic Kidney Disease Epidemiology Collaboration (CKD-EPI) formula as per the Australasian Creatinine Consensus Working Group’s position statements [18]. Similarly, HbA1c measures performed up to 30 days prior to the baseline visit were accepted, otherwise research assistants performed blood collection during the baseline visit. All HbA1c measurements were reported in both International Federation of Clinical Chemistry and Laboratory Medicine (IFCC) units (mmol/mol) and National Glycohaemoglobin Standardisation Programme (NGSP) units (%). These pathology measurements were undertaken at different laboratories based on General Practice clinic preferences. Masked CGM data was collected at baseline, prior to any therapeutic intervention, using a *FreeStyle Libre Pro®* sensor patch. This was applied by study staff to the underside of the participant’s upper arm to measure individual glucose levels in 15-min intervals for 2-weeks. After 2-weeks, the sensor was removed, and data were uploaded to a secure computer onto Microsoft Office Excel 2007 (Microsoft Corp., Seattle, WA, USA). Survey and clinical data were entered into REDCap© (REsearch Data CAPture software), a secure, web-based application designed to support research data capture [19].

### Measures

TIR, defined in this study as 3.9-10 mmol/l (70.2 – 180 mg/dL), is calculated using the CGM data. TIR was calculated using Microsoft Office Excel 2007 (Microsoft Corp., Seattle, WA, USA). The range of CGM data for inclusion in this study was 5 to 14 days, consistent with manufacturer’s recommendations [20].

We used the Socio-Economic Indexes For Areas (SEIFA) Index of Relative Socioeconomic Disadvantage (IRSD) as one measure of SES in our analysis. The SEIFA IRSD scores for each postcode are calculated by summarising attributes of the population collected through Australia’s national household census, such as income, educational attainment, employment and occupation. These scores are grouped into deciles where decile 1 represents the most disadvantaged and decile 10 represents the least disadvantaged [21].

The second measure of SES we used was the level of educational attainment. Educational levels were categorised into never attended, primary level, secondary level, Trade/Vocational training course (TAFE) and University diploma/degree.

Information on diet and exercise were obtained through a baseline questionnaire. Participants were asked to write down the number of days in the last week in which carbohydrates were evenly spaced, and the numbers of days in the last week in which ≥30 min of physical activity was undertaken.

### Data analysis

Normally distributed continuous variables are expressed as mean ± standard deviation, non-normally distributed continuous variables as median (IQ range) and categorical variables as frequency (percentage). Mixed-effects linear regression analysis was used to examine the four associations: TIR and IRSD, HbA1c and IRSD, TIR and educational attainment, and HbA1c and educational attainment. Never attended, primary education and secondary education were grouped into one category and used as a baseline to compare with other categories of education in our analysis. Univariable and multivariable analyses controlling for age, BMI, diet, exercise and study arm were performed to examine each association. Adjustment for study arm was performed as randomisation occurred after the CGM sensor was attached and before it was removed. Robust standard errors were specified to allow for clustering at the clinic level. As baseline HbA1c and IRSD deciles were non-normally distributed, log and square transformations of these variables were considered. Residual graphs were plotted for both transformed and untransformed data. Following review of the residual graphs, data transformation was not applied in our data analysis for all models of analyses. This is because the transformation did not significantly improve the random spread of the residuals, which results in limited benefit of the added complexity transformation would add to the interpretation. All statistical analyses were performed using STATA version 13.0 software (StataCorp, College Station, TX, USA).

### Ethics

The GP-OSMOTIC trial, incorporating this study, was approved by The University of Melbourne Human Research Ethics Committee (ID 1647151.3).

## Results

### Patient characteristics

Data from 278 of the 300 participants in the GP-OSMOTIC trial were included in this study. Ten participants were excluded from data analysis as their CGM data duration were < 5 days, thus limiting the accuracy of glucose profile output obtained from insufficient number of data points [20]. Three participants were excluded from this study’s analysis due to absent Socio-Economic Indexes for Areas (SEIFA) data based on the provided postcodes, and a further nine participants were excluded due to missing CGM data.

There was no difference in essential characteristics between included and excluded participants. Of the 22 participants excluded from this study, 10 (45.5%) were males, mean (SD) age was 57.4 (11.2) years, mean (SD) duration of T2D was 14.9 (4.6) years and mean (SD) BMI was 33.0 (4.6) kg/m^2^. Information obtained on diet and exercise were also similar with a median (IQR) of 4 (1,5) days and 5 (2, 6.75) days respectively.

The baseline characteristics of participants are summarised in Table 1.

**Table 1:** Demographic characteristics of 278 participants with T2D

Characteristics	n (%) (unless otherwise stated)	Missing data, n (%)
Male	167 (60.1)	–
Age in years^a^	61.0 ± 9.7	–
Country of birth		14 (5.0)
Australia	186 (70.5)	
Others^b^	78 (29.5)	
Healthcare Card Holder^c^	132 (49.4)	11 (4.0)
Private Health Insurance Owner	111 (41.6)	11 (4.0)
IRSD Decile^d^	4 (1, 6)	–
Education level		11 (4.0)
Never attended	1 (0.4)	
Primary	18 (6.7)	
Secondary	128 (47.9)	
Trade/TAFE	51 (19.1)	
University diploma/degree	69 (25.8)	
Employed	113 (42.3)	11 (4.0)
Smoking Status		12 (4.3)
Current Smoker	40 (15.0)	
BMI (kg/m^2^)^a^	34.0 ± 9.1	2 (0.7)
Diet^d,e^	3 (1, 5)	2 (0.7)
Exercise^d,f^	5 (3, 7)	2 (0.7)
Number of hypoglycaemic agents used		1 (0.4)
One agent	5 (1.8)	
Two agents	110 (39.7)	
Three agents	113 (40.8)	
Four or more agents	49 (17.7)	
Number of co-morbidities^d^	3 (2, 4)	–
Years of Diabetes^a^	14.3 ± 7.8	11 (4.0)
Diabetes-related Complications		
Micro-vascular	161 (57.9)	–
≥ 1 microvascular complication		
Macro-vascular	52 (18.7)	–
≥ 1 macrovascular complication		
Both micro- and macro-vascular complications	38 (13.7)	–
Duration of CGM use (days)^a^	12.3 ± 2.5	–
TIR(%)^a^	41.6 ± 25.4	–
HbA1c^d^		
%	8.6 (8.0, 9.7)	1 (0.4)
mmol/mol	70.5 (64.0, 82.5)	1 (0.4)
Intervention study arm	144 (51.8)	–

^a^Represents mean ± standard deviation

^b^Comprises Philippines, India, China, Singapore, Sri Lanka, East Timor, Afghanistan, Sudan, Timor, Indonesia, New Zealand, USA, Fiji and South Africa, UK, Poland, Former Yugoslavia, Malta, Northern Ireland, Italy, Poland and Netherlands

^c^Healthcare card holders are people in Australia who have a concession card provided by the Australian Government to enable them to get cheaper medicines and some healthcare cost discounts

^d^Represents median (IQ range)

^e^Represents number of days in the last week in which carbohydrates were evenly spaced

^f^Represents numbers of days in the last week in which ≥30 min of physical activity was undertaken

*IRSD* Index of Relative Socioeconomic Disadvantage, *BMI* Body Mass Index, *CGM* Continuous Glucose Monitor, *TIR* Percentage Time in Range, *HbA1c* Glycated Haemoglobin

### Association between TIR and IRSD

Table 2 shows the association between TIR and IRSD using unadjusted and adjusted models. There is evidence of a positive correlation between TIR and IRSD following adjustment for confounding variables. As the mean difference in TIR between one decile change of IRSD is 1.5% (95% CI 0.5, 2.5), thus on average, TIR was 15% higher for those least disadvantaged (IRSD = 10th decile) compared to those most disadvantaged (IRSD = 1st decile) (95% CI 5, 25).

**Table 2:** Association between TIR/HbA1c and IRSD/Education using unadjusted and adjusted models

Variable	Unadjusted	Adjusted^a^
TIR		
	Mean difference in TIR (95% Confidence Interval)	Mean difference in TIR (95% Confidence Interval)
IRSD Decile	1.7% (0.7, 2.6)	1.5% (0.5, 2.5)
Educational attainment		
Trade/TAFE	−5.7% (−16.2, 4.8)	−4.9% (−15.0, 5.3)
University/diploma	1.6% (−5.4, 8.7)	1.4% (− 5.5, 8.2)
HbA1c		
	Mean difference in HbA1c (95% Confidence Interval)	Mean difference in HbA1c (95% Confidence Interval)
IRSD Decile	−0.06% (− 0.1, − 0.01)	−0.06% (− 0.1, − 0.01)
Educational attainment		
Trade/TAFE	0.3% (− 0.06, 0.6)	0.2% (− 0.1, 0.6)
University/diploma	− 0.07% (− 0.3, 0.2)	−0.08% (− 0.4, 0.2)

^a^Multivariable analysis was adjusted for age, BMI, diet, exercise and study arm

### Association between baseline HbA1c and IRSD

Multivariable mixed effects linear regression identified an inverse correlation between HbA1c and IRSD. As the mean difference in HbA1c between one decile change of IRSD is 0.06% (95% CI 0.01, 0.1), thus on average, HbA1c was 0.6% lower for those least disadvantaged (IRSD = 10th decile) compared to those most disadvantaged (IRSD = 1st decile) (95% CI 0.1, 1).

### Association between TIR/HbA1c and education

Table 2 shows results of the association between TIR and educational attainment, as well as HbA1c and educational attainment following univariable and multivariable mixed effects linear regression. Educational attainment was not shown to be associated with either TIR or baseline HbA1c.

## Discussion

Our analysis of CGM data obtained over a 2-week period from participants with T2D and sub-optimal HbA1c in primary care as part of the GP-OSMOTIC trial provides novel insight into glycaemia in this population. Least disadvantaged IRSD deciles, a composite area level measure of socioeconomic disadvantage, were correlated with better glycaemic control (both TIR and HbA1c). Our results support that an increase of 5 deciles in IRSD was associated with bringing the mean TIR in this population to almost 50%. As having 50% of self-monitored glucose levels within 3.9-10 mmol/l(70.2 – 180 mg/dL) is correlated with an HbA1c of around 7% [12], this would be associated with significantly improved health outcomes and reduced risk of diabetes-related complications. However, we found no association between educational attainment, a single, individual level measure of SES, and glycaemic control (either TIR or HbA1c).

Our finding that educational attainment was not shown to be associated with achieving glycaemic targets despite the strong association seen for IRSD highlights the complexity of studying the concept of SES and its relationship with chronic disease parameters. Our finding is in contrast to international studies suggesting that socioeconomic advantage measured at the individual level such as higher educational attainment [22–25], higher income [26, 27] and higher residential stability [28], were associated with a greater likelihood of achieving HbA1c targets. However, there are many different measures and ways of defining SES, a concept that is made up of individual characteristics (e.g. educational attainment, income level, occupation), as well as contextual levels where the individual is situated within a physical and social location with characteristics relating to the built environment, social networks and social and supportive relationships. There is no single best indicator of SES suitable for all study aims and applicable at all stages in life. Rather there are many different possible measures, each with its own implications as well as strengths and limitations [29, 30]. It is thus important for policy, practice and research to be aware of this complexity, to use relevant SES measures and concepts that are suitable for their objectives and to interpret findings in relation to SES appropriately.

Individual and environmental factors likely interact with each other to affect glycaemia. People from more advantaged socio-economic backgrounds may have higher health literacy, with improved capacity to obtain, process, understand and act upon health information to support self-management of their condition [24]. However, our findings suggest that area level disadvantage is associated with glycaemia. Environmental and neighbourhood characteristics that we know are associated with more socio-economically advantaged areas such as greater accessibility to greenspace to engage in physical activities, a lower density of unhealthy food options such as fast food outlets, community norms and emphasis on healthy living and more opportunities to access health care services may all contribute to the social gradient in TIR and HbA1c that we identified [31].

It is important to acknowledge study limitations. The SEIFA IRSD represents an average of all people living in an area and does not represent individual situations. This is especially so in larger areas where there is more likely to be greater diversity [21]. The association between SES as measured by the IRSD and TIR is thus a generalised way of studying the link between neighbourhood level disadvantage with achievement of glycaemic targets. Other limitations of our study also include reliance on participants’ recall for certain information, such as duration of diabetes and the likelihood that T2D may have existed for months or even years before formal diagnosis. Information regarding participant medications, medical history and diabetes-related complications were retrieved from clinic medical electronic health records that may not be up to date. Our data were also sourced from only Victorian general practices and cannot be generalised to the whole Australian population. Lastly, heterogeneity in laboratory measurements (e.g. creatinine, albumin:creatinine ratio, HbA1c) which were performed in a variety of pathology laboratories may have weakened the correlation, though all laboratories would have been accredited and participate in a national quality control programme.

No studies have used TIR to look at the association with SES. As TIR may be a measure of glycaemic control that is easier for patients to understand, utilising TIR in clinical care particularly for patients from lower socioeconomic backgrounds may aid in increasing patient engagement, which in turn could assist in optimising self-care behaviours, improving health outcomes, and could contribute to reducing health inequities seen in T2D. Our findings, in conjunction with previous international [22–28] and Australian [5] studies, also have significant implications for resource allocation of community-based health services to reduce the health inequity gap in patients with T2D. It also aids in highlighting the importance for GPs to consider and understand patient context when engaging and supporting patients with their diabetes self-management. Further studies involving larger patient sample sizes over a longer period throughout Australia to investigate this association are warranted, as well as further studies investigating patient and practitioner perceptions of TIR as a measure of glycaemic control. Further studies of the relative effectiveness and cost of CGMs in this population would also be useful and will be conducted as part of the GP-OSMOTIC trial at a later stage.

## Conclusion

Our study showed a clinically and statistically significant association between an area-based measure of SES and glycaemic targets with more socio-economically disadvantaged people less likely to achieve glycaemic targets. Lower socioeconomic groups and areas may require prioritisation in resource allocation of primary health care services as well as policies aimed at ensuring equitable access to healthy environments to reduce health inequity. Our study highlights the importance of considering patient context during GP consultations and introduces a novel measure of glycaemia that may help with patient engagement to improve diabetes outcomes.

## Abbreviations

CGM: Continuous glucose monitor
GP-OSMOTIC: General Practice Optimising Structured MOnitoring To Improve Clinical outcomes (GP-OSMOTIC)
HbA1c: Glycated Haemoglobin
IRSD: Index of Relative Socioeconomic Disadvantage
SEIFA: Socio-Economic Indexes For Areas
SES: Socioeconomic Status
T2D: Type 2 Diabetes
TIR: Percentage Time in Range
